# Sex differences in gray matter volume: how many and how large are they *really*?

**DOI:** 10.1186/s13293-019-0245-7

**Published:** 2019-07-01

**Authors:** Carla Sanchis-Segura, Maria Victoria Ibañez-Gual, Jesús Adrián-Ventura, Naiara Aguirre, Álvaro Javier Gómez-Cruz, César Avila, Cristina Forn

**Affiliations:** 1 Departament de Psicologia bàsica, clínica i psicobiologia, Universitat Jaume I, Castelló, Spain; 2 Department of Mathematics. IMAC, Universitat Jaume I, Castelló, Spain

**Keywords:** Gray matter volume, Gender differences, Sex-sameness, TIV-adjustment, MRI, Effect size

## Abstract

**Background:**

Studies assessing volumetric sex differences have provided contradictory results. Total intracranial volume (TIV) is a major confounding factor when estimating local volumes of interest (VOIs). We investigated how the number, size, and direction of sex differences in gray matter volume (GMv) vary depending on how TIV variation is statistically handled.

**Methods:**

Sex differences in the GMv of 116 VOIs were assessed in 356 participants (171 females) without correcting for TIV variation or after adjusting the data with 5 different methods (VBM8 non-linear-only modulation, proportions, power-corrected-proportions, covariation, and the residuals method). The outcomes obtained with these procedures were compared to each other and to those obtained in three criterial subsamples, one comparing female-male pairs matched on their TIV and two others comparing groups of either females or males with large/small TIVs. Linear regression was used to quantify TIV effects on raw GMv and the efficacy of each method in controlling for them.

**Results:**

Males had larger raw GMv than females in all brain areas, but these differences were driven by direct TIV-VOIs relationships and more closely resembled the differences observed between individuals with large/small TIVs of sex-specific subsamples than the sex differences observed in the TIV-matched subsample. All TIV-adjustment methods reduced the number of sex differences but their results were very different. The VBM8- and the proportions-adjustment methods inverted TIV-VOIs relationships and resulted in larger adjusted volumes in females, promoting sex differences largely attributable to TIV variation and very distinct from those observed in the TIV-matched subsample. The other three methods provided results unrelated to TIV and very similar to those of the TIV-matched subsample. In these datasets, sex differences were bidirectional and achieved satisfactory replication rates in 19 VOIs, but they were “small” (*d* < ∣0.38∣) and most of them faded away after correcting for multiple comparisons.

**Conclusions:**

There is not just one answer to the question of how many and how large the sex differences in GMv are, but not all the possible answers are equally valid. When TIV effects are ruled out using appropriate adjustment methods, few sex differences (if any) remain statistically significant, and their size is quite reduced.

**Electronic supplementary material:**

The online version of this article (10.1186/s13293-019-0245-7) contains supplementary material, which is available to authorized users.

## Introduction

The subject of neuroanatomical sex differences in the brain is unique due to its scientific importance [1–4] and social repercussions [5, 6]. However, precisely quantifying sex differences in the volumes of specific brain regions is a challenging task, and studies assessing volumetric sex differences have provided heterogeneous and inconsistent results. Thus, for example, the right amygdala volume has been reported to be substantially larger in males (M > F [7], no different between females and males (F ≈ M [8]), and larger in females than in males (F > M [9]). The same thing occurs for many other gray and white matter structures (e.g., hippocampus: M > F [7], F ≈ M [8], F > M [9]; corpus callosum: M > F [10], F ≈ M [11–13], F > M [14]).

The inconsistencies and contradictions in the results of different studies evaluating volumetric sex differences are probably caused by many factors. However, it is believed that one of the major difficulties in these kinds of studies is that males and females differ in overall body and head size [11, 12, 15–17]. In other words, because sex differences in gross morphology may affect global and regional brain volumes, these differences introduce a major allometric challenge that might be subdivided into three hierarchically organized methodological questions.

First, the decision has to be made whether or not to adjust raw neuroanatomical volumes. This decision is quite important because unadjusted measures seem to affect the number and direction of sex differences in brain regional volumes [8, 9, 11–13, 16–19]. Nevertheless, there seem to be pros and cons of using both raw and adjusted volumetric measurements. Thus, adjusted brain measures are less reliable than unadjusted ones [20], but adjusted measures are currently considered more valid [21–23].

A second methodological decision refers to which variable should be chosen to adjust the gross morphological variations associated with sex. Several measures have been used for this purpose, including body weight, height, head circumference, total intracranial volume (TIV), and total brain volume. However, although they are still used by some researchers [24–26], body size parameters (such as height or weight) show weak and inconsistent correlations with overall brain size [27, 28], and they are generally perceived as inappropriate. The inadequacy of body size parameters as possible adjustment factors would be aggravated when trying to assess small regional volumes; therefore, total brain volume and TIV are usually preferred (for a more detailed discussion on this topic, see [29]).

Finally, after having decided to adjust their data and which adjustment factor to use (e.g., TIV), researchers must still choose from a variety of adjustment methods. Three methods (proportions, residuals, and covariate) have frequently been used to correct TIV scaling effects [30]. Two recent studies [16, 17] were specifically devoted to assessing whether the use of each of these adjustment methods affects the number and direction of brain volumetric sex differences. These studies showed that the use of proportion-adjusted data results in a larger number of sex differences, often indicating larger proportional gray matter volumes in females. By contrast, when using either of the other two methods, the number of sex differences is reduced, and their direction varies depending on the neuroanatomical region being considered. Therefore, evidence provided by these and other studies (e.g., [10, 31]) effectively confirmed that the choice of the TIV-adjustment method has a strong influence on the observed outcomes, thus showing its particular relevance in understanding the current lack of consensus about the number and direction of volumetric sex differences.

However, the studies by Nordenskjöld et al. [16], and Pintzka et al. [17], did not evaluate the outcomes when using two other currently available TIV-adjustment methods: the so-called power-corrected proportion adjustment method [15] and the one provided by the “non-linear only” modulation algorithm of the VBM8 [32]. Moreover, these two studies restricted their assessment to a short number of anatomical regions (*N* = 5 [16]; *N* = 18 [17]). Therefore, the present study was designed to confirm and extend the results of these studies by evaluating the results of five different TIV-adjustment methods in the 116 brain areas defined by the Automated Anatomical Labeling atlas (AAL [33]). More specifically, the aim of this study was fourfold. First, we aimed to assess to what extent sex differences in raw gray matter volumes are driven by TIV scaling effects. Second, we compared the number, size, and direction of the sex differences in the same 116  gray matter regional volumes after applying the five TIV-adjustment methods previously mentioned. Third, we tried to validate these methods by assessing (A) which of them satisfactorily removed TIV-scaling effects and (B) how their results compared to each other and to those obtained in three criterial subsamples. Fourth, we tried to summarize the most reliable differences by integrating the results obtained with the adjustment methods that were found to remove TIV effects.

We would like to note that the present study focuses on the statistical description of the possible female-male differences in gray matter volume but it does not assess whether or not they might have functional or behavioral consequences. We would also like to note that throughout this text, the term “sex” is used because this was the variable that the participants’ self-reported by choosing between two (male/female) categories. However, the use of this term does not imply any assumption on the possible origin of the observed differences (a topic that was not explored in the present manuscript, either).

## Materials and methods

### Participants and subsamples

For this study, we collected the scans of 356 healthy subjects (171 females; 185 males) who had participated in previous studies by our research team, recruited through local advertisements and word of mouth. All participants were physically and psychologically healthy, with no history of neurological or psychiatric disorders. The experiment was approved by the Ethical Committee of the University Jaume I (Spain).

The demographic characteristics of these participants are detailed in Table 1. In short, male participants were slightly older than female participants (*M* 22.39; SD 3.04 and *M* 21.64, SD 4.90, respectively), but this difference did not reach statistical significance. This effect was small (< 1 year), corresponding to Cohen’s *d* value 0.186 (that is, below of what Cohen defined as a small effect [34], p. 25–26), and unreliable (the 95% confidence intervals for the standardized and non-standardized difference between means included the zero value). On the other hand, female participants showed a wider age range but, as revealed by Levene’s test, the age variances of females and males did not significantly differ. Therefore, age was not considered a relevant variable in this study.

**Table 1:** Demographic characteristics of the participants included in the main sample and in the different subsamples used in the present study

	Main sample		Only-females		Only-males		TIV-matched	
	Males	Females	Large TIV	Small TIV	Large TIV	Small TIV	Males	Females
N	185	171	74	74	74	74	74	74
AGE (years)								
Mean (SD)	22.39 (3.04)	21.64 (4.90)	21.08 (2.76)	20.62 (2.98)	22.54 (3.05)	22.11 (3.13)	22.28 (2.97)	21.50 (2.71)
Range	18–30	18–49	18–30	18–30	18–30	18–30	18–30	18–31
Mean difference	0.75		0.46		0.43		0.78	
95% CI	[− 0.09, 1.59]		[− 0.47, 1.39]		[−0.57, 1.43]		[− 0.14, 1.70]	
Cohen’s *d* [95%CI]	0.18 [− 0.02, 0.39]		0.16 [− 0.16, 0.48]		0.14 [− 0.18, 0.46]		0.27 [− 0.05, 0.60]	
*t* test	*t*_*354*_ = 1.75, *p* = 0.08		*t*_*146*_ = 0.45, *p* = 0.27		*t*_*146*_ = 0.85, *p* = 0.39		*t*_*146*_ = 1.68, *p* = 0.10	
Levene’s test	*F* = 1.00, *p* = 0.36		*F* = 0.57, *p* = 0.45		*F* = 0.06, *p* = 0.85		*F* = 0.79, *p* = 0.37	
Education (years)								
Mean (SD)	14.61 (2.21)	14.56 (2.10)	14.61 (1.95)	14.61 (1.94)	14.69 (1.99)	14.55 (2.26)	14.69 (2.28)	14.62 (1.87)
Range	8–20	8–19	10–19	8–19	11–18	8–19	8–19	12–19
Mean difference	0.05		0.00		0.14		0.07	
95% CI	[− 0.40, 0.50]		[− 0.63, 0.63]		[− 0.55, 0.83]		[− 0.61, 0.75]	
Cohen’s *d* [95%CI]	0.02 [− 0.18, 0.23]		0.00 [− 0.32, 0.32]		0.06 [− 0.26, 0.39]		0.03 [− 0.29, 0.36]	
*t* test	*t*_*354*_ = 0.24, *p* = 0.81		*t*_*146*_ = 0.00, p = 1		*t*_*146*_ = 0.39, *p* = 0.70		*t*_*146*_ = 0.19, *p* = 0.84	
Levene’s test	*F* = 2.24, *p* = 0.13		*F* = 0.21, *p* = 0.65		*F* = 0.23, *p* = 0.63		*F =* 2.59, *p* = 0.11	

No statistically significant differences (*p* < 0.05) were observed for the age means (t tests) or variances (Levene’s test) of the compared groups in the main sample or in any of the criterial subsamples

The majority of participants (96.35%) were or had been university students (education years > = 12), and no differences were observed between females and males. As shown in Table 1, the unstandardized mean’s difference between females and males in this variable equated to 0.05 education years, and the standard deviations of both groups of participants were also very similar (2.10 and 2.21, respectively). Consequently, educational level was not considered a relevant variable in the present study.

From the participants’ pool, a “main sample” and 3 “criterial subsamples” were created.

#### Main sample

The “main sample” included the scanning data from all 356 participants, and it was employed to assess possible sex differences in gray matter volume in the unadjusted (hereinafter referred as raw) and TIV-adjusted datasets (see sections “Image pre-processing” and “TIV-adjustments methods”).

#### Criterial subsamples

Three criterial subsamples were constructed to provide independent estimations of the effects of sex (“TIV-matched” subsample) and TIV (“only female” and “only male” subsamples).

##### TIV matched subsample

The TIV-matched subsample was created by pairing each subject with the subject of the other sex with the nearest TIV, but only if this difference was ≤ 10 ml [17]. A total of 74 pairs of TIV-matched participants were created, resulting in two highly similar groups and a total subsample of 148 subjects. The demographic characteristics of the participants included in this subsample are detailed in Table 1.

“TIV-matching” is an artificial approach that excludes many participants, thus reducing data comparison to a TIV limited range and promoting a reduction in statistical power that might increase the chance of false negatives [16]. However, matching is the only undisputed method to completely remove head-size variation [31], and the results obtained in TIV-matched subsamples have been considered to be the best approximation to the “ground truth” of between-group (sex) differences [17].

##### Only-male and only-female subsamples

To directly test the effects of the TIV on gray matter volume, an “only-male” subsample and an “only-female” subsample were constructed (the demographic characteristics of the participants included in these two subsamples are detailed in Table 1). Each of these two single-sex samples was composed of one “large TIV” group and one “small TIV” group. To create these groups, participants of each sex were sorted in ascending order by their TIVs and median split into two equally sized participant pools. Seventy-four participants were first randomly selected from each participant pool, and the difference in the TIV averages of the resulting groups was calculated. Then, random within-pool replacements and between-pool permutations were iterated over these initial groups until they exhibited TIV differences similar to what was observed between the females and males in the main sample (≈*d* = 1.6; see the “Sex differences in gray matter volume: raw data” section). In this way, comparing the large/small TIV groups of the “only-female” and “only-male” subsamples provided sex-independent estimations of the TIV effects operating in the main sample. In this regard, it should be noted that, although the standardized size of the difference (Cohen’s *d*) between the large/small TIV groups of the only-male and only-female subsamples was the same (and matched what was observed between males and females in the main sample), the TIV range for the former (1360.49–1895.36) was larger than for the latter (1324.06–1641.79). This difference resulted in smaller averages, standard deviations, and *t* ratios for the large/small TIV groups in the only-female subsample than for their counterparts in the only-male subsample (see Additional file 1: Tables S9 and S10).

On the other hand, as the only male and only female subsamples were designed to have the same number of participants (74 + 74 = 148) and, therefore, the same statistical power as the TIV-matched subsample, the number of between-group differences in the three criterial subsamples could be directly compared. This made it possible to ascertain whether the TIV or the sex factor was able to produce a larger number of differences, and which of them mediated most in the differences observed in the main sample.

### MRI acquisition

MRI data were collected on a 1.5 T Siemens Avanto scanner (Erlangen, Germany). Anatomical 3D MPRAGE volumes were acquired using a T1-weighted gradient echo pulse sequence (TE, 3.8 ms; TR, 2200 ms; flip angle, 15°; matrix, 256 × 256 × 160 mm; voxel size, 1 mm^3^).

### Image pre-processing

Except in the case described in the section VBM8 non-linear modulation, images were preprocessed with the CAT12toolbox (http://www.neuro.uni-jena.de/cat/, version r1184) of the SPM12 (http://www.fil.ion.ucl.ac.uk/spm/software/spm12/, version 6906) software.

CAT12 preprocessing was conducted following the standard default procedure suggested in the manual. Briefly, this procedure includes the following steps: (1) segmentation of the images into gray matter, white matter, and cerebrospinal fluid; (2) registration to a standard template provided by the International Consortium of Brain Mapping (ICBM); (3) DARTEL normalization of the gray matter segments to the MNI template; (4) modulation of the normalized data via the “affine + non-linear” algorithm; and (5) data quality check (in which no outliers or incorrectly aligned cases were detected). Images were not smoothed because we were only interested in the modulated images.

Note that this procedure does not include any correction for overall head size (e.g., TIV correction).

Voxels were mapped into 116 regions according to the Automated Anatomical Labeling atlas (AAL [33]) by calculating the total gray matter volume for each region and participant via a MATLAB script (http://www0.cs.ucl.ac.uk/staff/g.ridgway/vbm/get_totals.m). This initial output (hereinafter, labeled as “raw” data) provided a volumetric dataset in which sex differences were evaluated and where all the TIV adjustment methods (except the one described in VBM8 non-linear modulation section) were applied. In addition, also following the standard CAT12 procedure, the total intracranial volume (TIV) was calculated as the sum of the gray matter, white matter, and cerebrospinal fluid volumes obtained in the tissue class images in native space.

### TIV-adjustment methods

With the exception of the VBM8-method, all TIV adjustments were implemented using SPSS 23 (IBM Corp.), PRISM 7.0 (GraphPad Inc.), and R, using as input the previously described raw CAT12 output.

#### VBM8 non-linear modulation

Until the recent development of the CAT12 software, VBM8 was probably one of the most popular programs for analyzing structural neuroimaging data. The VBM8 toolbox is a series of extensions to the segmentation algorithm implemented in the “New Segment” toolbox of the SPM8 (http://www.fil.ion.ucl.ac.uk/spm/software/spm8/) software.

In this study, the so-called optimized voxel-based morphometry (VBM) protocol [35] was used to automatically obtain gray matter volumes corrected for individual TIV size (hereinafter, referred to as “VBM8-adjusted dataset”). The image preprocessing was carried out with the VBM8 toolbox (version r445) under SPM8 (version 6316). Similarly to the CAT12, this protocol includes five main steps: (1) segmentation of the images into gray matter, white matter, and cerebrospinal fluid; (2) registration to a standard template provided by the International Consortium of Brain Mapping (ICBM); (3) a high-dimensional DARTEL normalization of the gray matter segments to the MNI template; (4) non-linear modulation (a step in which the normalized gray matter segments are multiplied only by the non-linear determinants of the normalization deformation matrix to correct the images for individual differences in size [32]; and (5) data quality check (in which no outliers or incorrectly aligned cases were detected). Finally, following the same procedure described in the “Image pre-processing” section for the CAT12, we also calculated the total gray matter volume of the 116 AAL regions from the modulated images of each participant.

To isolate the effects of the TIV-adjustment introduced by the non-linear modulation step and ensure that the outcomes of the VBM8-adjusted dataset were fully comparable to those of all the other adjustment methods, a second set of VBM8 images was obtained. In this case, VBM8 images were preprocessed following the same protocol described above, but the images were modulated using the “affine + non-linear” algorithm, which does not correct for individual differences in brain size. Sex differences were also calculated in this uncorrected “affine + non-linear VBM8” dataset and compared to those observed in the CAT12 raw dataset (Additional file 1: Table S2).

#### Proportion adjustment method

This method implicitly assumes a proportional relationship between TIV and the volume of any neuroanatomical structure of interest (VOI). The adjusted volume (VOI_adj_) is individually calculated according to the following formula:

$$ {\mathrm{VOI}}_{\mathrm{adj}}=\mathrm{VOI}/\mathrm{TIV} $$

Therefore, the resultant is not an absolute quantity, but rather a ratio or proportion, and the adjustment operates at the individual level (although it might be averaged by group, and between-group differences might be determined using difference tests; O’Brien et al. [29]).

#### Covariate regression method

This procedure does not provide adjusted VOIs that are free of TIV-scaling effects. Instead, it allows estimating the group (in this case, sex) effects without any influence of the TIV effect, by simultaneously introducing TIV and sex as putative predictors of each VOI in a multiple regression model, resulting in the following formula:

$$ \mathrm{VOI}={\mathrm{b}}_0+{\mathrm{b}}_{\mathrm{TIV}}\mathrm{TIV}+{\mathrm{b}}_{\mathrm{sex}}\mathrm{sex}+\upvarepsilon $$

This method incorporates information from all the participants, and having a similar number of participants in each group (sex) becomes critical to ensure the reliability of the results [16]. In addition, because all the parameters included in the regression model compete in explaining the variance in each VOI, the obtained standardized regression coefficients (β_TIV_ and β_sex_) already provide a direct estimation of the variation that can be associated with the TIV and sex for each VOI. Moreover, each regression coefficient is associated with a significance level, thus making second-level analyses (i.e., between-group difference tests) unnecessary. Finally, as the unstandardized *b*_sex_ coefficients represent the average predicted difference between males and females for each VOI while all other independent variables are held constant, Cohen’s *d* can be estimated by dividing the b coefficients obtained by the corresponding VOIs’ standard deviations.

#### PCP

The power-corrected proportion method (PCP) was recently proposed by Liu et al. (2014) as an improvement over the commonly used “proportion method” (see the “Proportion adjustment method” section). This method explicitly assumes that the relationship between the TIV and a VOI is not proportional, but instead follows a power law. Thus, corrected volumes are estimated through a VOI/TIV ratio that includes an exponential correcting parameter, leading to the generic formula:

$$ {\mathrm{VOI}}_{\mathrm{adj}}=\mathrm{VOI}/{\mathrm{TIV}}^{\mathrm{b}} $$

The *b* parameter of this formula was obtained by calculating the slope value of the regression line between LOG(VOI) and LOG(TIV).

#### The residuals adjustment method

This procedure was initially discussed by Arndt et al. [20], but its use spread after its reevaluation by Mathalon et al. [21]. This method aims to remove an implicitly assumed linear TIV-VOI relationship through the following formula:

$$ {\mathrm{VOI}}_{\mathrm{adj}}=\mathrm{VOI}\hbox{-} \mathrm{b}\left(\mathrm{TIV}\hbox{-} \overline{TIV}\right), $$

where *b* is the slope of the VOI-TIV regression line, and $\overline{TIV}$ is the mean of the TIV measures of the *control group*. When, as in the study of sex differences, there is no control group, the VOI-TIV regression and the $\overline{TIV}$ are calculated using the whole sample of participants.

### Statistical analyses

#### Sex differences

Except for the covariate regression adjustment method (see “Covariate regression method” section), sex differences in gray matter volume were assessed through 116 separate Student’s *t* tests for independent groups. The significance threshold was initially set at 0.05, although when describing the results for the criterial subsamples (whose size is less than half of that of the main sample), differences that achieve *p* values below 0.1 are also mentioned in the main text, and exact *p* values for all comparisons are provided in the corresponding Supplementary Tables. To maximize statistical power, no corrections for multiple comparisons were initially introduced, and following recent recommendations of the American Statistics Association [36, 37], we focused our analysis on effect sizes rather than *p* values. Nevertheless, in a separate section (“Replication of differences across methods”), we assessed how different multiple-comparison correction methods (two false discovery rate and two family-wise error) changed the number of statistically significant differences observed in each TIV-adjusted dataset. More specifically, in decreasing order according to their expected statistical power, the Benjamini, Krieger and Yekutieli [38] Benjamini and Hochberg [39], Holm [40] and Bonferroni-Dunn [41] corrections for multiple comparisons were tested.

Furthermore, effect sizes were estimated by calculating Cohen’s *d* values and their corresponding 95% confidence intervals (CI). In this study, positive *d* values indicate larger gray matter volumes in males than in females (M > F), whereas negative *d* values indicate larger gray matter volumes in females than in males (F > M). Following recent recommendations [42–44], the Cohen’s *d* values for the most reliable sex differences (see the “Replication score” section) were transformed into two more intuitive effect size indexes: the percent of overlap and the percent of superiority [45]. The percent of overlap denotes the proportion of scores that overlap in two normal distributions which means differ in some magnitude, whereas the percent of superiority denotes the probability that a randomly sampled member of population *a* will have a score (*Y*_*a*_) that is higher than the score (*Y*_*b*_) attained by a randomly sampled member from population *b* [46]. These indexes were estimated using the online calculator provided by Magnusson, 2014 [47] at http://rpsychologist.com/d3/cohend/, which computes the percent of overlap using the rationale and amended proportions described in [48] and the percent of superiority described in [49].

#### Evaluation of the TIV-adjustment methods

##### Relationship with the TIV before and after TIV adjustment

Previous studies have shown that in the absence of any correction, the local volumes of particular brain areas are directly related to the TIV [15, 17, 18, 29]. The presence of this relationship in our own raw data was assessed by performing linear regression analyses relating the TIV and each of the 116 VOIs considered in this study. The possible effects of these predicted linear TIV-VOI relationships on the observed sex differences in gray matter volumes were also investigated by calculating the rank-order correlation between the slope values of the former and the *p* and Cohen’s *d* of the latter. Because females and males differ in TIV, larger sex differences would be more likely in VOIs showing a steeper relationship with TIV.

TIV-VOI_adj_ relationships provided a first and powerful criterion to evaluate the goodness of the different adjustment methods tested in this study. That is, because the aim of the adjustment methods is to get rid of TIV effects and provide an unadulterated estimation of sex differences, satisfactorily adjusted data should not show the linear TIV-VOI_adj_ relationship predicted for the raw data, and the likelihood or size of sex differences in local gray matter volumes should not be associated with TIV-VOI_adj_ slope values. Therefore, deviations from zero in the slope values of the 116 TIV-VOI_adj_ regression lines, as well as their possible rank order correlation with the *p* and Cohen’s *d* values of the sex differences observed, were assessed in each TIV-adjusted dataset. In addition, when adequate, chi-squared association tests were used to compare the relative frequency of sex differences in the brain regions showing significant/non-significant linear relationships with TIV.

##### Concordance between methods

The degree of agreement in the methods was initially assessed at the nominal (statistically significant difference/no statistically significant difference) level using the free-marginal multi-rater kappa index [50, 51]. Moreover, following the directions provided by O′ Brien et al. [30], the overall agreement across methods was also assessed in terms of ordinal ranking through Kendall’s W. Finally, and also following the methodology described by O′ Brien et al. [30], we used Spearman’s rho correlation to specifically compare the concordance between each pair of methods. In these analyses, *p* values were used instead of test statistics because the former provide standardized versions of the latter that can be compared across all the adjustment methods and samples used in the present study (for a more detailed discussion, see [30]).

##### Relationship with criterial subsamples

Spearman’s rho was used to quantify the similarity between the *p* values of the between-group differences observed in the criterial subsamples and the sex differences obtained in the raw and TIV-adjusted datasets.

To obtain a more detailed comparison with the TIV-matched subsample, we analyzed the relative frequency of coincidental and non-coincidental findings of this criterial subsample and each TIV-adjusted dataset. A coincidental result (hit) was scored when (1) a statistically significant sex difference of the same sign was found in the same anatomical region in a TIV-adjusted dataset and in the TIV-matched subsample; or (2) when a statistically significant sex difference in a particular brain region was neither found in the TIV-adjusted dataset and in the TIV-matched subsample. On the other hand, non-coincidental results (no-hits) included (1) “false positives” (when a statistically significant sex difference found in a TIV-adjusted dataset was not replicated in the TIV-matched subsample); (2) “false negatives” (when a statistically significant sex difference found in the TIV-matched subsample was not observed in a TIV-adjusted dataset); and (3) “reversions” (when statistically significant differences of an opposite sign were found in the TIV-matched subsample and in a TIV-adjusted dataset). These data were analyzed by means of Cohen’s kappa agreement index, codifying statistically significant M > F differences as 1, non-statistically significant differences as 0, and statistically significant F > M differences as − 1. The Cohen’s kappa values obtained were interpreted according to the guidelines provided by Landis and Koch [52], which define “poor” (kappa < 0.0), “slight” (0.00–0.20), “fair” (0.21–0.40), “moderate” (0.41–0.60), “substantial” (0.61–0.80), and “almost perfect” (0.81–1.00) levels of agreement.

##### Replication score

Trying to identify the brain areas where sex differences might have the highest and lowest likelihood of occurring, a replication score was calculated. This calculation was carried out using the results obtained in the TIV-matched subsample, as well as with results from adjusted datasets that proved to be trustworthy. More specifically, attending to the codification of Cohen’s *d* sign used in the present study (see the “Relationship with the TIV before and after TIV adjustment” section), M > F statistically significant differences were scored as + 1, F > M statistically significant differences were scored as − 1, and the absence of statistically significant differences was scored as 0. In a second step, the individual scores for each VOI in the different datasets were added together, and the final score obtained was interpreted without attending to its sign. A difference was considered highly replicable when it was observed in all or all except one of the included data sets. In addition, taking into account that the absence of evidence does not necessarily provide evidence of absence [53], a more restrictive criterion (replication score = 0) was applied before concluding “sex sameness” or a consistent lack of sex differences.

## Results and discussion

### Sex differences in gray matter volume: raw data

#### Sex differences: number and size

**Fig. 1:**
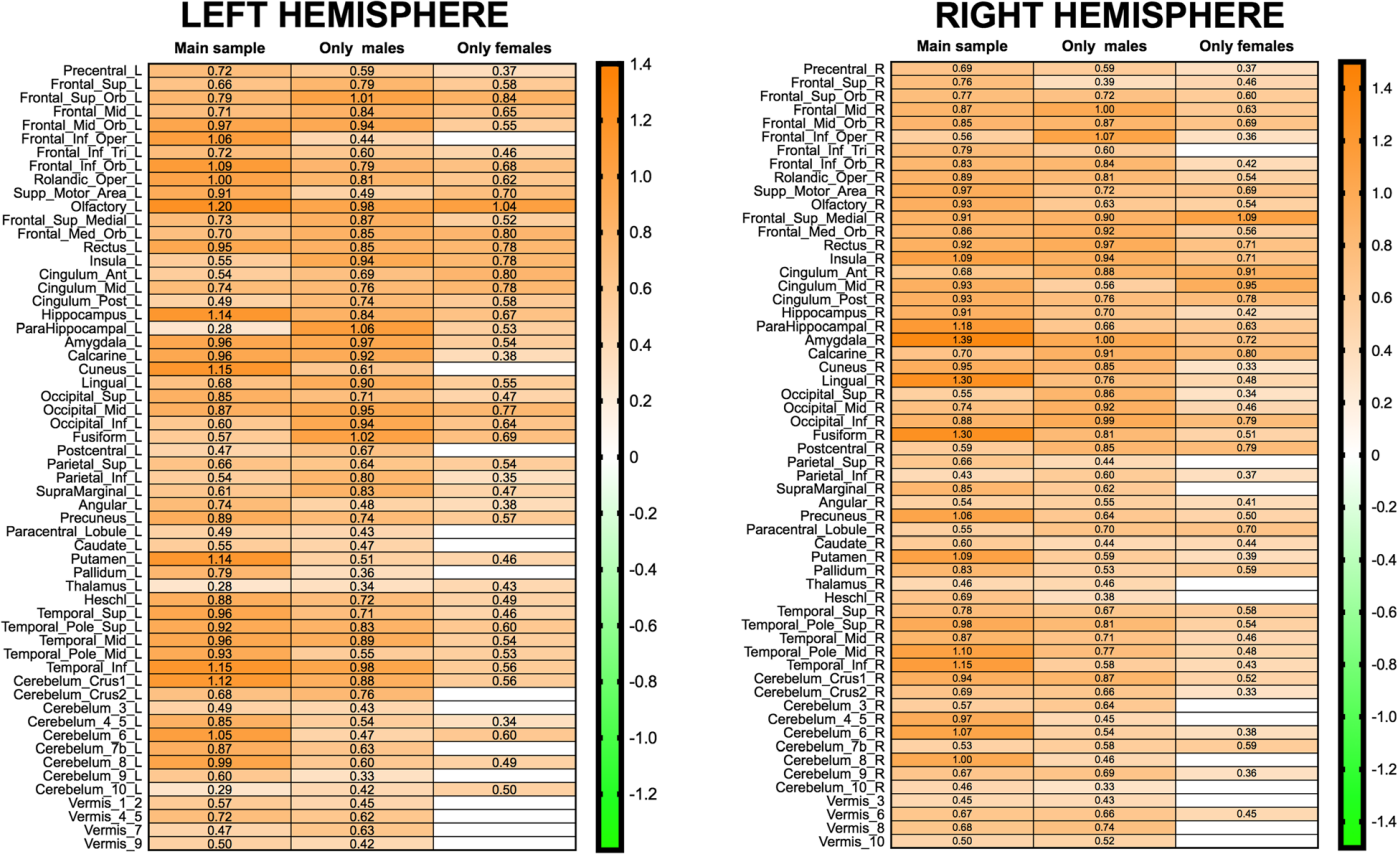
Effect sizes of between-group differences in the main sample and in the “only-males” and “only-females” subsamples. Panels left and right present odd and even numbered brain anatomical regions of the AAL atlas, which (with the exception of the lobules of the cerebellar vermis) are located in the left and right hemisphere, respectively. Each column of this heatmap displays the Cohen’s *d* values for statistically significant (*p* < 0.05, uncorrected) between-group differences found in each sample (effect sizes of non-significant differences are found in Additional file 1: Tables S1, S9 and S10). Orange and green correspond to effects favoring the groups with larger/smaller TIV (which in the case of the main sample were males/females), respectively

**Fig. 2:**
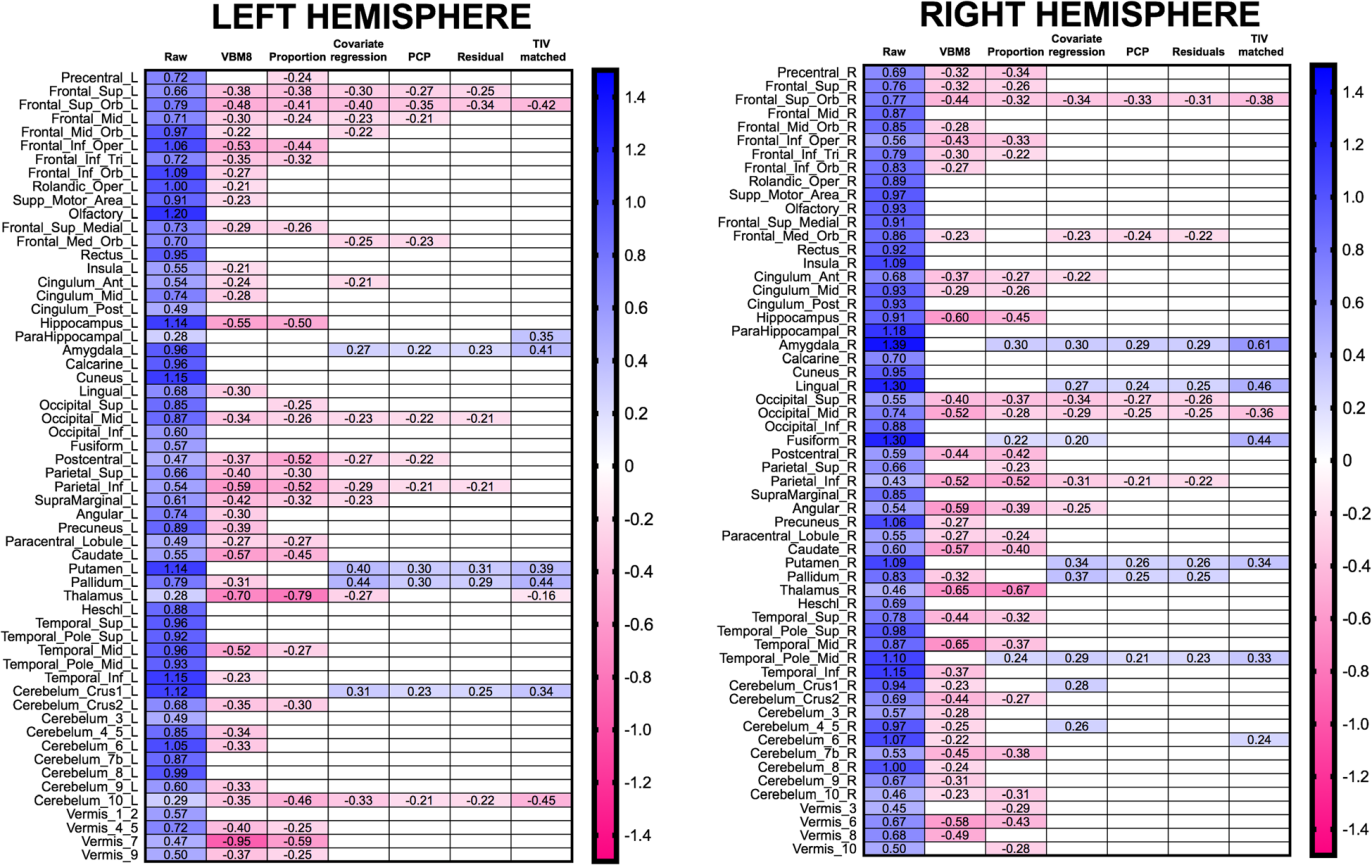
Effect sizes of sex differences in each dataset. Panels left and right present odd and even numbered brain anatomical regions of the AAL atlas, which (with the exception of the lobules of the cerebellar vermis) are located in the left and right hemisphere, respectively. Each heatmap displays the Cohen’s *d* values for statistically significant (*p* < 0.05, uncorrected) sex differences found in each dataset (effect sizes of non-significant differences are found in Additional file 1: Tables S1 and S3-S8). Blue and red correspond to M > F and F > M effects, respectively

Males had larger total intracranial volumes than females [TIV; *t*_354_ = 15.05, *p* < 1^−15^; Cohen *d* = 1.596 (95% CI 1.357, 1.835)]. Statistically significant differences were also found for each volume of interest (VOI), with males exhibiting larger gray matter volumes than females in all cases (see details in Additional file 1: Table S1A). As Figs. 1 and 2 show, the size of these effects ranged from 0.279 (#77, Thalamus_L) to 1.390 (#42, Amygdala_R), with an average of 0.811 (95% CI: 0.770, 0.852).

These results are highly similar to those from previous studies assessing the total gray matter and local volumes in pre-selected neuroanatomical areas [11–13, 17, 18].

#### Sex differences: relationship with TIV

**Fig. 3:**
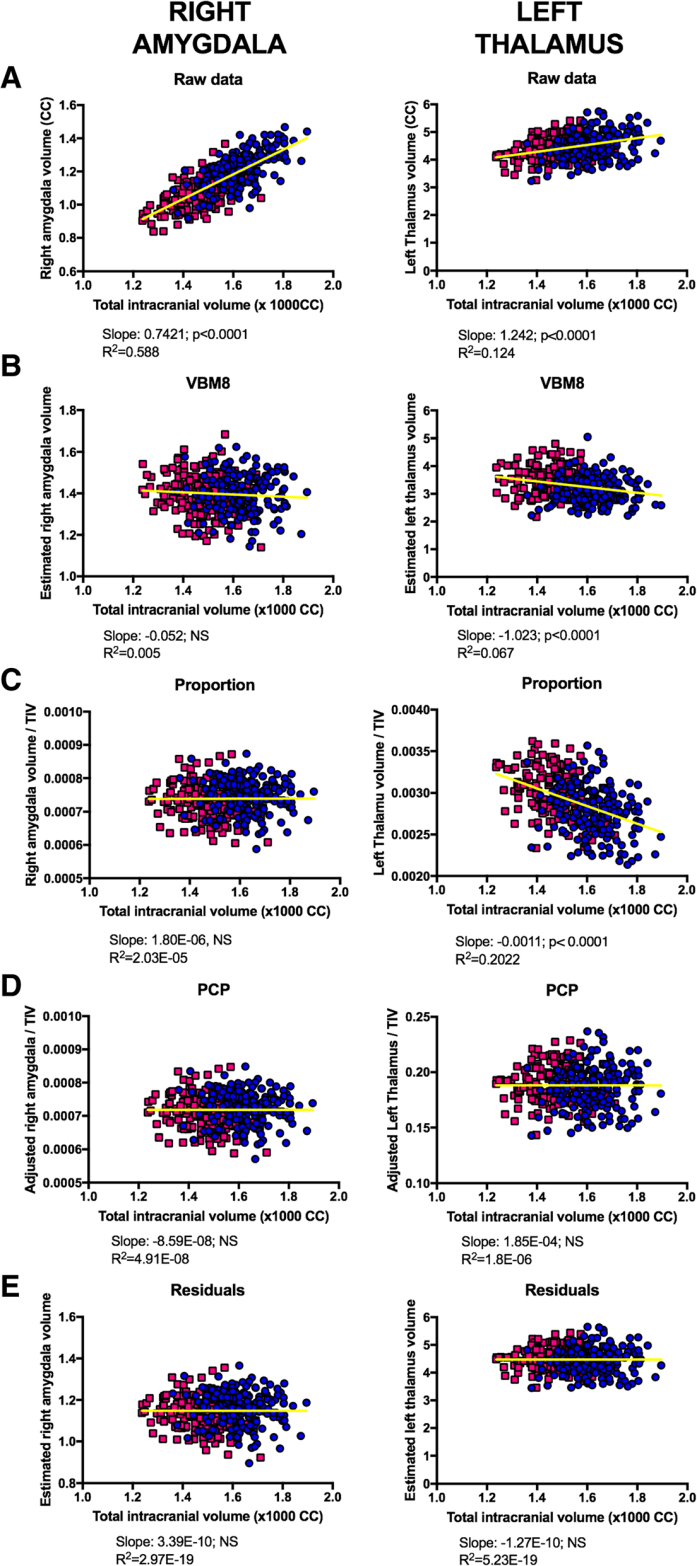
TIV-VOI relationships in raw and TIV-adjusted datasets. Scatterplots and outcomes of linear regression analyses of the raw or VBM8-, proportion-, PCP- or residual-adjusted volumes of the right amygdala (right), or the left thalamus (left) vs. intracranial volume are shown. This regression analysis was conducted on the 116 regions of the AAL atlas, and its output is fully reported in Additional file 1: Tables S1, S3, S4, S6, S7, and S8

Previous studies have shown that the raw volumes of several brain anatomical structures are directly, but not uniformly related to TIV [11, 15, 17, 18, 31, 54]. We replicated and extended these previous findings by quantifying the direct and linear relationship between TIV and each of the 116 VOIs defined in the AAL atlas. Thus, as exemplified in Fig. 3a and fully described in Additional file 1: Table S1B, the strength of the TIV-VOI relationships was generally high, but not uniform across brain areas. More specifically, the percent of variance accounted for by TIV ranged from 9.60 (#115, Vermis_9) to 59.82 (#56, Fusiform_R) and averaged 37.10% (95% CI 34.6, 39.5). The distinct percent of variance explained by TIV at each VOI was partly explained by the different sizes of these regions, with TIV accounting for larger amounts of variance in anatomical regions with larger average volumes (Pearson’s *r* = 0.471, *p* < 1.59^− 8^).

The slopes of these VOI-TIV linear relationships also showed wide variation across different brain areas, ranging from 0.042 (#109, Vermis_1_2) to 11.510 (#8, Frontal_Mid_R), with an average of 3.228 (95% CI 2.787, 3.669). As predicted (see “Relationship with the TIV before and after TIV adjustment” section), the steepness of these TIV-VOI relationships, along with the sex differences in TIV, fueled sex differences in local gray matter volumes. Indeed, the TIV-VOI slope values were correlated with both the significance level (Spearman’s rho − 0.414, *p* < 0.0001) and the size (Spearman’s rho 0.423, *p* < 0.0001) of the observed sex differences in local gray matter volumes. These results verify that the higher the TIV, the higher the gray matter volume in each VOI. More importantly, these results also show that the tighter the TIV-VOI relationship, the larger and more likely the sex differences, thus revealing that differences between females and males in raw gray matter volume are at least partially dependent on TIV scaling effects.

#### Comparison with criterial subsamples

The large- and small-TIV groups in the only-male subsample differed in their TIV [*t*_146_ = 9.962, *p* < 1^−15^; Cohen’s *d* = 1.653 (95% CI 1.372, 1.934)] and in the 116 VOIs considered in this study (Additional file 1: Table S9A). In all cases, the large-TIV group had larger local gray matter volumes than the small-TIV group (*L* > *S*; see Fig. 1), with an average *d* of 0.701 (95% CI 0.665, 0.736). As expected, both the effect sizes (Spearman’s rho 0.359, *p* < 0.0001) and significance levels (Spearman’s rho − 0.359, *p* < 0.0001) of these differences were significantly correlated with the slope of the 116 TIV-VOI regression lines (Additional file 1: Table S9B). Similarly, the large- and small-TIV groups in the only-female subsample differed in their TIV [*t*_146_ = 9.61, *p* < 01^−15^; Cohen’s *d* = 1.650 (95% CI 1.370, 1.930)]. As shown in Fig. 1, local volumetric differences (*L* > *S*) with *p* values below 0.05 were observed in 90 brain regions [average *d* = 0.571 (95% CI 0.536, 0.605)], and *L* > *S* differences with *p* values below 0.1 were observed in 12 more VOIs (Additional file 1: Table S10A). As expected, the significance level (Spearman’s rho − 0.370, *p* < 0.0001) and size (Spearman’s rho 0.368, *p* < 0.0001) of these differences were correlated with the slope of the 116 TIV-VOI regression lines (Additional file 1: Table S10B). Taken together, these results reveal that, in the absence of any effects of sex, a TIV difference of the same magnitude as the one observed in the main sample results in widespread and medium-to-large local volume differences that unfailingly favor the groups with larger TIVs.

On the other hand, the females and males in the TIV-matched subsample had virtually identical TIVs [*M*_females_ 1545.111, SD 77.372; *M*_males_ 1546.191, SD 75.397; *t*_146_ = 0.086, *p* = 0.931; Cohen’s *d* = 0.01; 95% CI − 0.308, 0.336]. Local volumetric differences attained *p* values below 0.05 in 15 brain regions (and below 0.1 in 12 more; Additional file 1: Table S8A). As shown in Fig. 2, males exhibited larger VOIs in 11 (73.33%) anatomical regions [average *d* = 0.405 (95% CI 0.351, 0.459)], and females exhibited larger VOIs in 4 cases [average *d* = − 0.402 (95% CI − 0.337, − 0.466)]. This striking decrease in the number of statistically significant sex differences (− 87% compared to the main sample) could initially be due not only to effective removal of the TIV effects, but also to a reduction in statistical power derived from the smaller size of the TIV-matched subsample. However, several sources of evidence provide support to the former possibility: (1) a similar reduction (− 80%) in the number of sex differences was also observed in the TIV-matched subsample of Pintzka et al. [17], which was almost as large as our main sample (*N* = 304 and *N* = 354, respectively); (2) despite having the same size and statistical power, more numerous and larger between-group differences were observed in our only-male and only-female subsamples; (3) the reduction in sample size cannot account for the reduction (− 76.68%) or the change in direction of the effect sizes of more than half (10 out of 19; 52.63%) of the differences observed in our TIV-matched subsample. Therefore, the low number, the reduced size, and the bidirectionality of the sex differences observed in the TIV-matched subsample is due to removal of TIV effects and not to its reduced statistical power. Accordingly, neither the significance levels nor the effect sizes of the sex differences observed in this subsample were correlated (Spearman rho 0.046, *p* = 0.619 and 0.136, *p* = 0.143, respectively) with their corresponding TIV-VOI slope values (provided in Additional file 1: Table S8B).

From the results obtained in our criterial subsamples, it became apparent that “sex differences” in the main sample were more similar (in number, average size, and direction) to the differences observed between the large/small-TIV groups in the only female and only male subsamples than to the sex differences observed in the TIV-matched subsample. This qualitative conclusion was validated by a correlational analysis. Thus, the *p* value ordering of these sex differences was much more correlated with the *p* value ordering of the differences observed between the large/small TIV groups of the only-female (rho = 0.547, *p* < 1^−8^) and only-male (rho = 0.500, *p* < 1^−8^) subsamples than with those corresponding to the male-female differences in the TIV-matched subsample (rho = 0.257, *p* < 0.01). Indeed, the *p* value ordering of the “sex differences” in the main sample correlated almost as much with those of the only-male and only-female subsamples as the latter two did with each other (rho = 0.600, *p* < 1^−12^).

These results confirm that raw gray matter volumes of females and males conflate sex and TIV-scaling effects, and they suggest that the latter might be quantitatively more important (a conclusion confirmed by other results from the present study, see “Covariate regression” section). Therefore, most sex differences observed in the raw gray matter volumes of unselected females and males seem to result from TIV-scaling effects, making it necessary to remove the effects of TIV before evaluating any possible specific sex differences in gray matter volume.

### Sex differences in gray matter volume after TIV adjustment: number and size

As expected, TIV-adjustment reduced the number and size of sex differences in gray matter volume. However, as described below, the number, size, and direction of these sex differences were strikingly dependent on the method used to correct for the TIV effects.

#### VBM8-adjusted dataset

As expected, when using the “affine + non-linear VBM8” algorithm (which does not correct for TIV variation), sex differences were observed in each of the 116 brain areas defined by the AAL atlas. These differences (Additional file 1: Table S2) were very similar in direction (all M > F) and size (range 0.215–1.51; average 0.900) to those observed in the raw dataset obtained with CAT12 preprocessed images.

By contrast, after applying the VBM8 “non-linear only” modulation algorithm to correct for individual differences in TIV (VBM8-adjusted dataset), statistically significant sex differences were found in just 71 VOIs. In all cases, females exhibited larger VBM8-adjusted gray matter volumes than males (for a complete statistical output, see Additional file 1: Table S3A). As depicted in Fig. 2, the effect sizes of these differences ranged from − 0.210 (#29, Insula_L) to − 0.949 (#113, Vermis_7), with an average of − 0.383 (95% CI − 0.417, − 0.350).

#### Proportion adjusted dataset

When using proportion-adjusted data, statistically significant sex differences were found in 51 adjusted VOIs (Additional file 1: Table S4A). As Fig. 2 shows, in 48 cases (92.15%), females exhibited larger proportional volumes than males, and the effect sizes of these differences ranged from − 0.785 (#77, Thalamus_L) to − 0.222 (#14, Frontal_Inf_Tri_L), with an average of − 0.359 (95% CI − 0.393, − 0.323). Males exhibited larger proportional volumes than females in only three regions (#42, Amygdala_R; d = 0.296; #56, Fusiform_R; d = 0.216; #88, Temporal_Pole_Mid_R; *d* = 0.244).

#### Covariate regression

When TIV and sex were simultaneously included in a multiple linear regression analysis, sex became a relevant predictor of 31 VOIs (for a complete statistical output, see Additional file 1: Table S5). As Fig. 2 shows, in 19 cases (61.29%), females exhibited larger VOIs than males. The effect sizes of these differences ranged from − 0.213 (#31Cingulum_Ant_L) to − 0.397 (#5, Frontal_Sup_Orb_L), with an average of − 0.273 (95% CI − 0.249, − 0.298). In the 12 cases where males had larger VOIs than females, the effect size of the differences ranged from 0.201 (#56, Fusiform_R) to 0.439 (#75, Pallidum_L) and averaged 0.310 (95% CI 0.269, 0.352).

In a different vein, it is worth noting that, whereas sex was only a relevant predictor of 31 VOIs, TIV was a significant predictor in all of the 116 VOIs considered in this study. Moreover, the standardized regression coefficients (β) corresponding to the TIV (*M* 0.600, SD 0.132) were significantly larger than those for sex (*M* − 0.003, SD 0.092; *t*_115_ = 33.41; *p* < 0.0001; Cohen’s *d* = 5.33; see Additional file 1: Table S5). Accordingly, the semi-partial correlations corresponding to TIV (*M* 0.468, SD 0.103) were higher (*t*_115_ = 53.76, *p* < 0.0001; Cohen’s *d* = 5.08) than those for sex (*M* − 0.0025; SD 0.072). Once again, these results indicate that most sex differences in raw gray matter volumes are actually driven by TIV-scaling effects, hence confirming the findings and conclusions of the “Sex differences in gray matter volume: raw data” section.

#### PCP adjustment method

The calculated *b* parameter varied widely across the different regions of interest (range 0.430, 1.155; average 0.863; see Additional file 1: Table S6A). When these *b* values were used to adjust the TIV-based proportions, significant sex differences were found in 22 VOIs (for a complete statistical output, see Additional file 1: Table S6A). In 13 cases (59.09%), females had larger power-corrected proportion (PCP)-adjusted gray matter volumes than males, with effect sizes ranging from − 0.211 (#7, Frontal_Mid_L) to − 0.351 (#5, Frontal_Sup_Orb_L); average − 0.247 (95% CI − 0.219, − 0.275). In the other 9 cases (M > F), effect sizes ranged from 0.214 (#88, Temporal_Pole_Mid_R) to 0.301 (#73, Putamen_L), with an average of 0.257 (95% CI 0.232, 0.283). The anatomical localization of all these sex differences is shown in detail in Fig. 2.

#### Residual adjustment method

When using the residual adjustment method, 19 VOIs showed statistically significant differences between females and males (for a complete statistical output, see Additional file 1: Table S7A). As Fig. 2 shows, in 10 cases (52.63%), females exhibited larger gray matter residual-adjusted volumes, and the effect sizes of these differences ranged from − 0.210 (#51, Occipital_Mid_L) to − 0.343 (#5, Frontal_Sup_Orb_L), with an average of − 0.248 (95% CI − 0.215, − 0.280). In the 9 cases where males had larger residual-adjusted VOIs than females, the effect sizes ranged from 0.226 (#88, Temporal_Pole_Mid_R) to 0.306 (#73, Putamen_L), and their average was 0.261 (95% CI 0.239, 0.284).

### Evaluation of the adjustment methods

#### Relationship between TIV and adjusted VOIs

As introduced in the “Relationship with the TIV before and after TIV adjustment” section, the main goal of the adjustment methods tested in this study is to remove any influence of TIV scaling effects. Therefore, in contrast to what was observed in raw VOIs (“Sex differences: relationship with TIV” section), properly adjusted VOIs should not show any significant linear relationship with TIV, and the likelihood and size of the sex differences observed in these adjusted VOIs should be unrelated to the slope values obtained when calculating these regression lines. These predictions were tested in the VBM8-, the proportion-, the PCP-, and the residuals-adjusted datasets (but not for the outcomes of the covariate-regression method because it does not produce adjusted VOIs; see the “Covariate regression method” section), but they were only confirmed in the last two.

Thus, applying the VBM8 “non-linear only” modulation algorithm reduced the strength and, in most cases, inverted the direction, but it did not eliminate the TIV-VOI_adj_ relationship (see Fig. 3b and Additional file 1: Table S3B) or its effects on sex differences. More specifically, we observed that the slope values of the 116 regression TIV-VOI_adj_ lines were significantly correlated with the significance levels (Spearman’s rho 0.555, *p* < 0.0001) and effect sizes (Spearman’s rho 0.574, *p* < 0.0001) of the sex differences in these VBM8-adjusted VOIs. These slope values were significantly different from zero in 52 anatomical regions, and sex differences were more frequently observed [*χ2* (1, *N* = 116) = 12.35, *p* = 0.0004] in them (41/52; 78.84%) than in the regions non-significantly related to TIV (30/64; 46.87%).

Similarly, the proportion adjustment method reduced the strength and, in most cases, inverted the direction, but it did not remove all the TIV-VOI_adj_ linear relationships (Fig. 3c and Additional file 1: Table S4B). A remaining and inverted relationship between TIV and proportion-adjusted local gray matter volumes had been previously reported [11, 15, 19, 21], but its relevance for the number and size of sex differences had not been explored. In this regard, and parallel to what was observed in the VBM8-adjusted data, the 116 slope values of the TIV-VOI_adj_ regression lines were significantly correlated with the significance levels (Spearman’s rho 0.366, *p* < 0.0001) and effect sizes (Spearman’s rho 0.541, *p* < 0.0001) of the sex differences in these proportion-adjusted VOIs. These slopes were significantly different from zero in 63 proportion-adjusted VOIs (Fig. 3c and Additional file 1: Table S3), and most of the sex differences were observed in these anatomical regions [38/63, 60.31% vs. 13/53, 24.52%; *χ2* (1, *N* = 116) = 14.97, *p* < 0.0001].

Conversely, adjusting the VOIs by means of the PCP or the residuals methods completely eliminated their relationship with TIV (see Fig. 3d, e and Additional file 1: Tables S6B and S7B). Thus, none of the regression lines between TIV and PCP- or residual-adjusted VOIs differed significantly from zero. Moreover, the slopes of these regression lines did not show any statistically significant relationship with the significance levels or the effect sizes of the sex differences observed in PCP- (Spearman’s rho − 0.051, *p* = 0.585; Spearman’s rho 0.168, *p* = 0.070) and residual- (Spearman’s rho 0.051, *p* = 0.585; Spearman’s rho 0.102, *p* = 0.271) corrected VOIs, respectively.

Taken together, these results reveal that VBM8- and proportion-adjusted data remain related to TIV and, although their effects operate in an inverse direction to what was observed in the raw data (“Sex differences in gray matter volume: raw data” section), they have an influence on the sex differences observed in these datasets. However, the sex differences observed in PCP- and residual-adjusted data (as well as those estimated from covariate regressions) are free of any influence of TIV.

#### Agreement across methods

As revealed by the free-marginal multi-rater kappa concordance index, there was a poor to modest level of nominal (statistically significant difference/non-statistically significant difference) agreement among the methods (*Κ* = 0.32; 95% CI 0.23–0.42; estimated overall agreement 66.21%). Similar results and conclusions were obtained when concordance was assessed at the ordinal level through Kendall’s coefficient of concordance (*W* = 0.408, *p* < 0.0001). Spearman correlations (Table 2) revealed that these modest levels of agreement actually result from merging two separate “clusters” of outcomes. Thus, the ordering of the *p* values obtained in the VBM8-adjusted dataset was similar to the one obtained in the proportion-adjusted dataset (Spearman’ s rho 0.695, *p* < 5^−18^, but unrelated to those obtained when data were adjusted with any other method (which were virtually identical among them; Spearman’ s rho values ranging from 0.980 to 1, *p* < 1^−80^). The only exception to the sharp separation of these two clusters of methods was a weak (rho = 0.195, *p* < 0.05) correlation between the *p* value ranks of the proportion and the PCP methods.

**Table 2:** Concordance between the sex differences obtained in each adjusted dataset

	VBM8	Proportion	Covariate regression	PCP	Residuals
VBM8	–	0.695**	0.047	0.091	0.047
Proportion	0.695**	–	0.110	0.195*	0.110
Covariate regression	0.047	0.110	–	0.981***	1.000***
PCP	0.091	0.195*	0.981***	–	0.981***
Residuals	0.047	0.110	1.000***	0.981***	–

Spearman’s rho rank correlations were calculated using the *p* value ordering for each pair of adjustment methods (*p < 0.05, **p < 5^−18^, ***p < 1^−80^). The *p* values used in these calculations were obtained in the male-female VOI comparisons in each TIV-adjusted dataset

#### Relationship with criterial subsamples

As Table 3 shows, the *p* value orderings of the sex differences observed in the VBM8- or proportion-corrected data were correlated with the between-group differences observed in the only-male/only-female subsamples and in the raw dataset, but they were only marginally (*r* < 0.18, p ≈ 0.06) correlated with the sex differences found in the TIV-matched subsample. Conversely, the *p* value orderings of the sex differences observed in the covariate regression-, the PCP- or residual-adjusted datasets were highly and exclusively correlated with those observed in the TIV-matched subsample (*r* > 0.64, *p* <  1^-8^in all cases). These results confirm and extend the results of the “Relationship between TIV and adjusted VOIs” section by indicating that the sex differences observed in VBM8- and proportion-adjusted datasets are probably more related to TIV-scaling than to sex effects. Therefore, it might be concluded that, only in the covariate regression-, PCP and residual-corrected datasets, and unbiased estimates of sex effects might be obtained.

**Table 3:** Correlations between sex differences in each adjusted dataset and the between-group differences in the criterial subsamples

	VBM8	Proportion	Covariate regression	PCP	Residuals
TIV-matched (sex effect)	− 0.177^$^	− 0.179^$^	0.722***	0.648***	0.722***
Only males (TIV effect)	− 0.211*	− 0.205*	0.085	0.103	0.085
Only females (TIV effect)	− 0.250**	− 0.241**	0.059	0.070	0.059
Raw (TIV and sex effects)	− 0.529***	− 0.640***	− 0.022	− 0.064	− 0.022

Correlations between the *p* values of the sex differences obtained in each adjusted dataset and the *p* values of the between-group differences observed in the three criterial subsamples. Spearman’s rho rank correlations were calculated using the ordering of the *p* values of the sex differences obtained in each adjusted dataset and the group effects observed in criterial subsamples, providing unbiased estimations of sex (TIV-matched subsample) and TIV (only-males and only-females subsamples) effects. For comparison purposes, the correlations with the *p* values of the sex differences observed in raw gray matter volumes are also provided (^$^*p* < 0.06, *p < 0.05, **p < 0.01, ***p < 0.1^−8^)

**Fig. 4:**
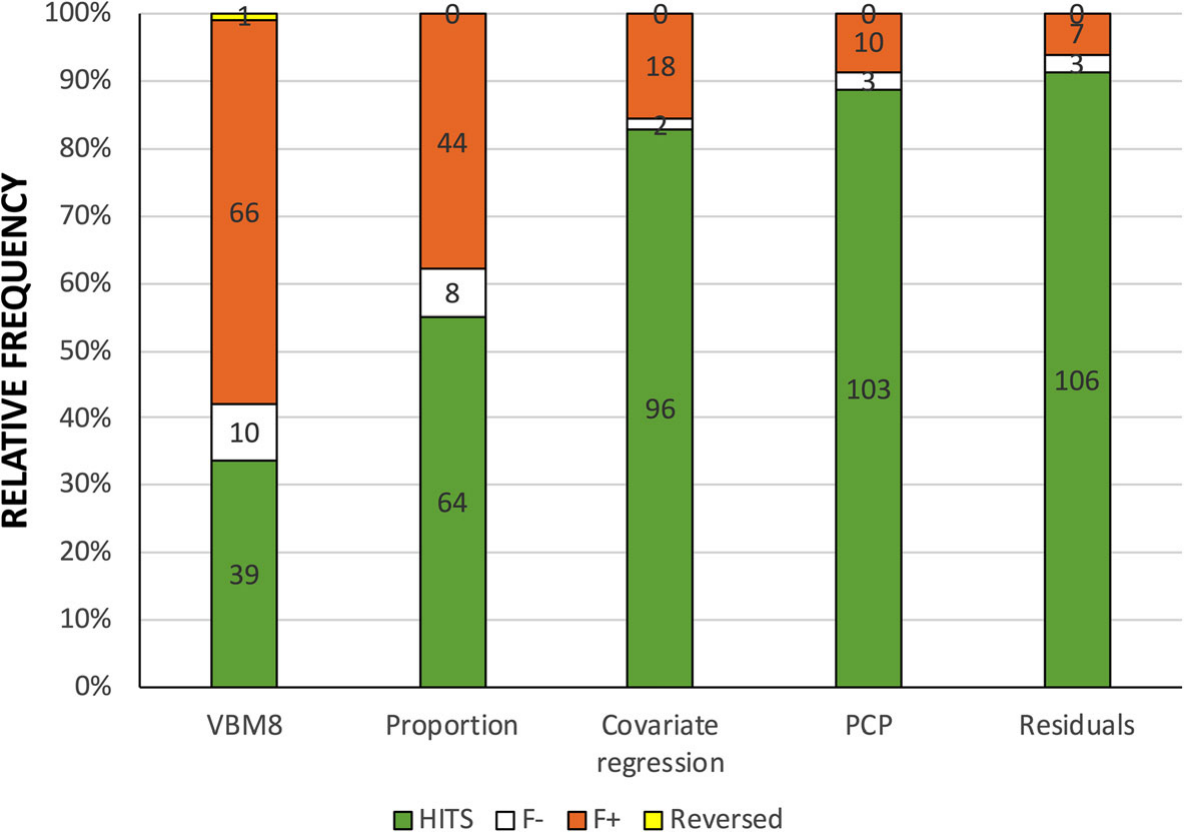
Comparison with the TIV-matched sub-sample. This Figure summarizes the relative (%, *Y* axis) and absolute frequencies (numbers within the bars) of coincident (hits, green) and non-coincident (“false negatives,” white; “false positives,” orange and “reversed” differences, yellow) results of each adjusted dataset and those observed in the TIV-matched subsample

A more detailed comparison of the results obtained in each adjusted dataset and those obtained in the TIV-matched subsample was conducted using the Cohen’s kappa concordance index (Fig. 4). Interestingly, the level of agreement in the outcomes of the TIV-matched and VBM8-adjusted datasets was not different from what would be expected by chance (κ = − 0.035; 95% CI − 0.095, 0.025; *p* = 0.270), and similar results were observed when considering the proportion-adjusted dataset (κ = 0.095; 95% CI − 0.020, 0.210; *p* = 0.030). However, the outcome of the covariate regression method (κ = 0.502, 95% CI 0.324, 0.680; *p* = 1^−15^) showed levels of agreement with the TIV-matched subsample that might be considered moderate. Moderate but very close to the boundary of “substantial” (κ = 0.61) agreement was observed in the PCP-adjusted dataset (κ = 0.604; 95% CI 0.413, 0.795, *p* = 1^−18^), whereas the residuals-adjusted dataset ( κ = 0.670; 95% CI 0.483, 0.857; *p* = 1^−20^) surpassed this threshold and showed the highest degree of agreement with the TIV-matched subsample.

### Reliability of the differences

#### Replication of differences across methods

As described in the “Relationship with the TIV before and after TIV adjustment” section, to identify the most consistent sex differences and sex similarities, a replication score was calculated. This score only took into account the outcomes of datasets adjusted with methods that are free of TIV effects (the covariate regression-, the PCP-, and the residuals-adjusted datasets).

A consistent lack of sex differences (replication score = 0) was observed in 83 of the 116 VOIs (71.55% of total; see Additional file 1: Table S11). However, as Table 4 shows, consistent sex differences (replication scores ≥ 3) were identified in 19 VOIs (10 F > M; 9 M > F; 16.4% of total). The *d* values for these differences ranged between |0.2–0.6| depending on the VOI and adjustment method considered. The confidence intervals of the estimated effect sizes were relatively broad, thus indicating that the precision of these estimates is suboptimal. Moreover, in some cases, confidence intervals included the zero value, which introduces some uncertainty about the reliability of these differences. On the other hand, when the *d* values for each VOI were averaged across methods, these effect sizes became smaller and varied within a narrower range (*d* = |0.22–0.38|).

**Table 4:**
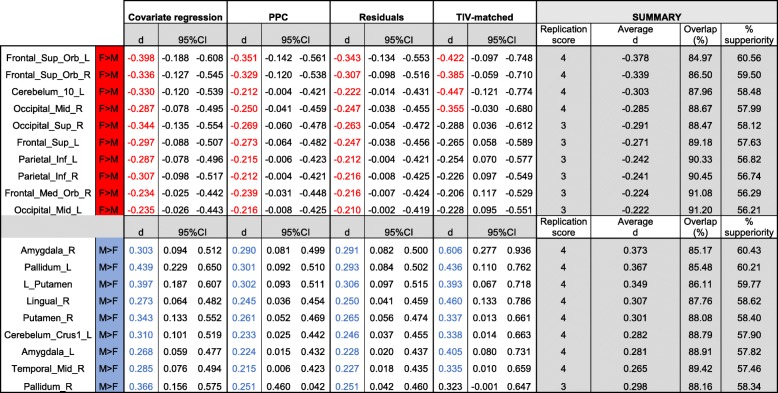
Summary of sex differences

Cohen’s *d* values and the lower and upper limits of the 95%confidence intervals of the sex differences with replication scores > 3(see details in the “Replication score” section) are provided. *d* values in red/blue correspond to sex differences favoring females/males with uncorrected *p* < 0.05 (values in black correspond to differences with *p* > 0.05). The average *d* was calculated by incorporating all these *d* scores (*p* < 0.05 and *p* > 0.05), and this value was used to compute the percentage of overlap of females and males and the percent of superiority

#### Effects of multiple comparison correction

Up to this point, all the effects presented in this study assumed a significance threshold (*p* < 0.05) that did not account for a large number of comparisons performed. This methodological decision was made to maximize statistical power and reduce type II errors, but it increases the probability of type I errors (see “Sex differences” section). Therefore, we sought to investigate how several procedures to correct for multiple comparisons affected the number of statistically significant sex effects in each TIV-adjusted dataset, as well as in the raw volumetric data.

**Fig. 5:**
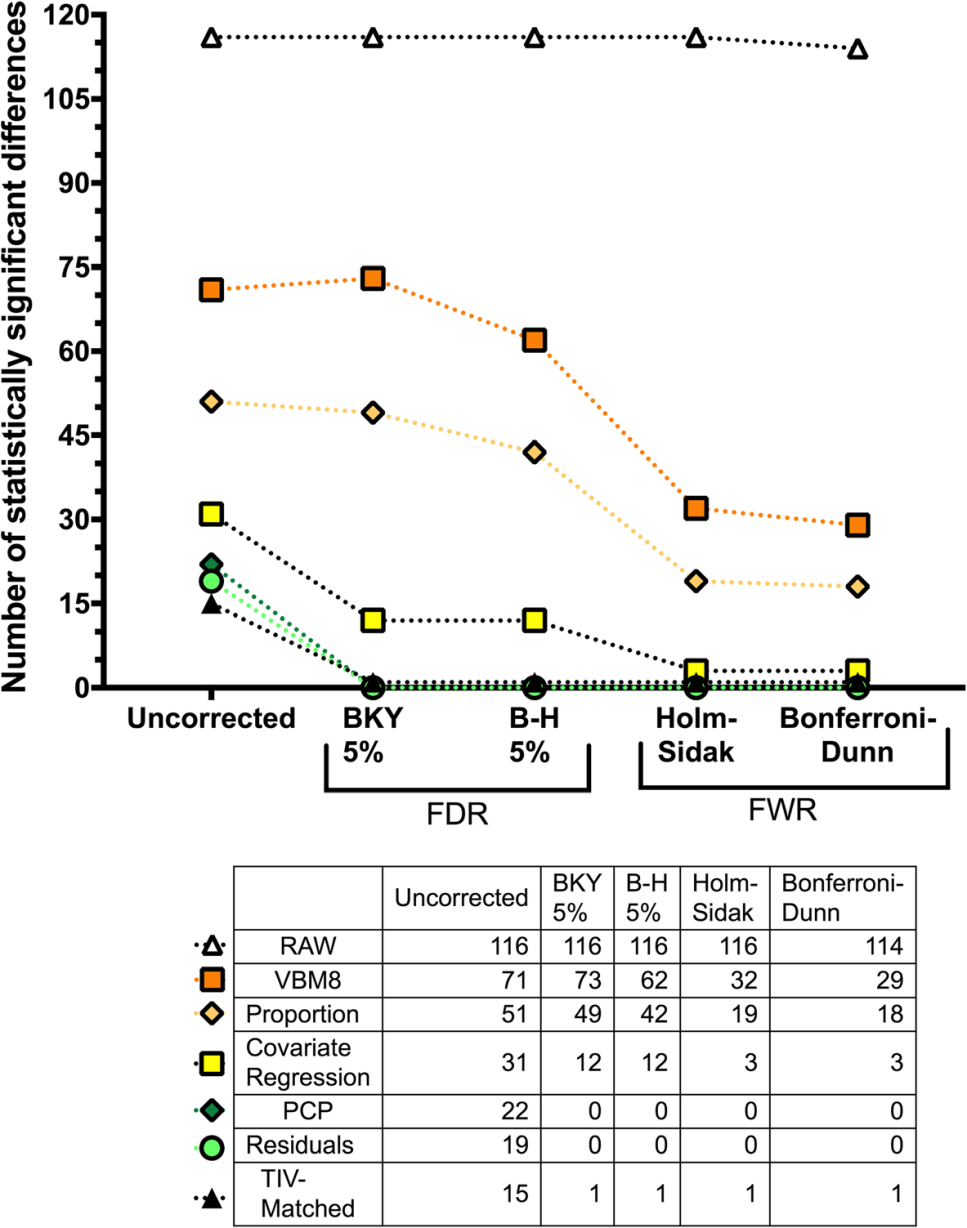
Effect of different procedures to correct for multiple comparisons on the number of sex differences in raw and TIV-adjusted datasets. FDR, false discovery rate; FWR, family-wise error rate; BKY, Benjamini, Krieger, and Yekutieli; B-H, Benjamini and Hochberg

As Fig. 5 shows, correcting for multiple comparisons resulted in a reduction in the number of statistically significant differences. This effect was more pronounced in the PCP- and residuals-adjusted datasets, in which even the most liberal correction procedures resulted in levels of significance above 0.05 for each VOI. A similar decrease was observed in the TIV-matched subsample, although the sex difference observed in the right amygdala retained statistical significance across all the correction procedures. On the other hand, the decline in the number of statistically significant differences was less sharp in the covariate regression—and even less so in the proportion and the VBM8-adjusted datasets. Moreover, in the VBM8-adjusted dataset, adopting Benjamini-Krieger-Yekeuteli-corrected *p* values resulted in a larger number of statistically significant differences than when using uncorrected *p* values (a paradoxical effect that is not uncommon in studies involving between-group comparisons of brain structure measures [55]). Finally, the number of differences observed in the raw dataset was mostly unchanged, and only when using the Bonferroni-Dunn correction, two (out of 116) comparisons failed to reach statistical significance.

These results reinforce the concerns about the reliability of some of the sex differences arising from the inspection of confidence intervals (“Replication of differences across methods” section). The possible causes and interpretations of these findings are further discussed in the “Discussion and conclusions” section.

## Discussion and conclusions

The results of the present study allow us to draw three main conclusions. First, most male-female differences in regional gray matter volumes are due to sex-independent TIV-scaling effects. Therefore, these female-male differences are not “sex differences,” but rather “size differences.” Consequently, it is necessary to remove the effects of TIV when trying to evaluate any possible sex effects on local gray matter volumes.

Second, not all methods currently used to remove TIV variation are equally effective and valid. Thus, choosing an appropriate adjustment procedure becomes a critical methodological decision that should be reported in detail and carefully considered when summarizing the results of different studies. In this regard, although none of these methods can be designated as “the correct one” [29], our results indicate that the proportion- and the VBM8 (“non-linear only modulation”) adjustment methods invert, but do not remove, the effects of TIV, hence producing patterns of sex differences that are opposite to, but just as misleading, as the ones provided by raw data. As a result, these two adjustment methods should probably be abandoned (for similar conclusions, see [16, 17, 20, 29, 54]). However, the other three methods evaluated here effectively remove TIV effects (“Relationship between TIV and adjusted VOIs” section; Table 3). Although the outcomes of these methods are very similar (Table 2), the ones obtained from the residuals- and PCP-adjusted datasets showed a slightly higher degree of concordance with those from the TIV-matched subsample than the outcomes obtained with the covariate regression method (Fig. 4). Nevertheless, the higher flexibility of this method might recommend its use in particular circumstances (e.g., when it is necessary to incorporate additional covariates; see [29]). Therefore, choosing one of these three valid methods should be guided more by the sample characteristics, the measures that are available, and the experimental design than by any a priori recommendation (for a more comprehensive discussion, see [16, 29, 30].

Third, when TIV effects are properly controlled, sex differences in gray matter volumes seem to be relatively infrequent and small. However, a precise and definitive answer to the question of how many and how large the sex differences in gray matter volume are cannot be provided.

In any case, the question of how many sex differences there are might be considered spurious because statistical significance (whether or not a consensual but arbitrary probability threshold is surpassed) does not equate to scientific relevance, and because statistical significance (and, thereby, the number of differences found) is critically dependent on sample size. Plainly speaking, with a large enough sample, any discrepancy becomes a “statistically significant difference” [56], but such a finding might be more informative about the sample than about the difference itself [10, 57, 58]. Indeed, as recently mentioned in a statement by the American Statistical Association [36], *p* values have no inferential content, and they do not measure the size or the importance of a result. Therefore, following the recommendations of the ASA and other similar claims [10, 59–62], the emphasis should be on estimation rather than testing, and effect size information should replace “bright-line” interpretations of *p* values. For the present study and other similar studies, this means focusing more on the size than on the number of sex differences. Nevertheless, it is worth mentioning that not only in this study, but also in others with larger sample sizes [11, 17, 19], the number of statistically significant sex differences is much lower than the number of sex similarities, especially when adopting a significance level corrected for multiple comparisons (Fig. 5).

According to Cohen’s cut-offs [34], the estimated effect sizes of the sex differences found in our study were “small” (Fig. 3). However, these effects exhibited relatively wide 95% confidence intervals (Table 4, Additional file 1: Tables S3–S7), especially in the TIV-matched subsample (Additional file 1: Table S8). This is the case because, although effect size measurements are independent from the sample size, the sample size affects the precision of their estimation [63]. Therefore, it might be argued that the *actual* effect sizes of the sex differences in cerebral gray matter volumes could be larger than those reported in our study. However, this seems unlikely because reduced sample size tends to overestimate, not to underestimate, the size of statistically significant effects (“the winner’s curse effect” [64];). Indeed, several studies [11, 17, 19, 65] using valid TIV-adjustment methods in samples larger than ours, estimated effect sizes that were similar, but smaller, than those provided here. This might be illustrated by using the amygdala volume as an example. Thus, our estimated average *d* values for the right and left amygdala (0.373 and 0.281, respectively; Table 4) were higher than the bilateral amygdala volumes estimated in other large residuals—or ANCOVA—TIV adjusted datasets ([65] *N* = 883, *d* = 0.25 [11]; *N* = 856, η^2^ = 0.011 ≈ *d* = 0.21 [17]; *N* = 998, *d* = 0.18 [19]; *N* = 2400, *d* = 0.18) and those estimated in a recent meta-analysis ( [66] right amygdala; Hedges *g* ≈ *d* = 0.171; left amygdala, Hedges *g* ≈ *d* = 0.233). Therefore, it might safely be concluded that the actual sizes of the sex differences in gray matter volumes should be similar to or smaller than those reported in our study, and that they are “small.”

Although initially appealing, Cohen’s “size-labels” for effect sizes (“small,” “moderate,” and “large”) are ambiguous in their meaning. Effect size meaning is better conveyed by *d*-derived indexes, such as the percent of overlap and the percent of superiority displayed in Table 4. These results clearly show that, even in the anatomical regions at which the largest sex differences were found, gray matter volumes present an impressive degree of overlap (ranging between 84.97 and 91.20%). Accordingly, the probability that a randomly sampled person from one sex will have a larger gray matter volume than a randomly sampled person from the other sex never exceeded the 60.56% (that is, just 10.56% more than what would be expected by chance). The meaning of this observation is better appreciated by comparing it to the size of the somatic male-female differences such those observed in as height, at which overlap is reduced to 31.66% and the percent superiority (in this case, M > F) raises up to the 92% [45]. Therefore, the effect sizes observed in this study clearly reinforce the notion that local gray matter volumes of females and males are more alike than different, and that none of their differences can be described as an example of “sexual dimorphism” (literally, “two forms”). Nonetheless, labeling the observed effects as “small” is not the same as saying that they are trivial. Small effects might be meaningful [42, 67]. Moreover, effect size interpretation is always dependent on the research context [68]. Thus, small sex differences such as those observed in the present study might become relevant in the context of psychiatric or neurological disorders, whereas they might be far less relevant in many other research contexts [69, 70]. However, whether or not this is the case remains to be demonstrated in future studies.

## Limitations

The present study has some limitations that reduce the generalizability of its results.

First, it should be noted that we used a convenience sample (rather than sampling epidemiological techniques) that covered a relatively narrow age range and was mainly composed of university students. Although these characteristics are typical of most volumetric studies in non-clinical populations, they may reduce generalizability to other populations.

Second, in this study, we employed a VOI-based approach using the AAL atlas. Although this approach has less anatomical precision than voxel-based analyses, it was chosen because (1) it defines the VOIs before conducting any data analysis, hence avoiding circularity and SHARKing and contributing to the accurate estimation of effect sizes [71, 72]; 2) It reduces the number of between-group comparisons, thus contributing to obtaining an adequate balance between sensitivity and statistical power. More specifically, after setting the power at 0.8 and assuming the conventional significance threshold of 0.05, the minimum detectable effect in this study was estimated as *d* ≥ 0.29. In this way, restricting the number of between-group comparisons to 116 predefined VOIs allowed us to detect even small effects while maintaining statistical power at much higher levels than those ordinarily observed in neuroimaging studies [64, 73]. However, it should be noted that, although the AAL is probably the most commonly used atlas in MRI studies, this atlas was constructed based on the neuroanatomical characteristics of a single brain [33], and it also presents other limitations inherent to the use of any predefined template [74].

## Additional file

Additional file 1: **Table S1.** A. Descriptive statistics (mean and SD) and sex-based volumetric comparisons in the raw dataset). B. TIV-VOI linear regression analyses. **Table S2.** Descriptive statistics (mean and SD) and sex-based volumetric comparisons in the VBM8 “affine + non-linear” dataset. **Table S3.** A. Descriptive statistics (mean and SD) and sex-based volumetric comparisons in the VBM8- non-linear only TIV-adjusted dataset. B. TIV-VOI linear regression analyses. **Table S4.** A. Descriptive statistics (mean and SD) and sex-based volumetric comparisons in the proportions-adjusted dataset. **Table S4.** B. TIV-VOI linear regression analyses. **Table S5.** A. Sex and TIV contribution to local gray matter volumes as quantified by the covariate regression method. **Table S6.** A. Descriptive statistics (mean and SD) and sex-based volumetric comparisons in the power-corrected proportion (PCP) TIV-adjusted dataset B. TIV-VOI linear regression analyses. **Table S7.** A. Descriptive statistics (mean and SD) and sex-based volumetric comparisons in the residuals TIV-adjusted dataset. **Table S8.** A. Descriptive statistics (mean and SD) and sex-based volumetric comparisons in the TIV-matched subsample. **Table S9.** A. Descriptive statistics (mean and SD) and sex-based volumetric comparisons in the “only males” subsample. B. TIV-VOI linear regression analyses. **Table S10.** A. Descriptive statistics (mean and SD) and sex-based volumetric comparisons in the “only females” subsample B. TIV-VOI linear regression analyses. Additional file 1: **Table S11.** A. Replication score. B Cohen’s *d* averages (XLSX 554 kb)

## Data Availability

The datasets containing the raw and adjusted data used during the current study are available from the corresponding author on reasonable request.

## Abbreviations

AAL: Automated Anatomical Labeling atlas
BH: Benjamini and Hochberg correction for multiple comparisons
BKY: Benjamini, Krieger, and Yekutieli correction for multiple comparisons
CAT12: Computational Anatomy Toolbox
CI: Confidence interval
F-: False negatives
F: Female
F +: False positives
FDR: False discovery rate
FWER: Family-wise error rate
GMv: Gray matter volume
M: Male
MRI: Magnetic resonance imaging
PCP: Power-corrected proportion
TIV: Total intracranial volume
VBM: Voxel-based morphometry
VOI: Volume of interest
VOI_adj_: Adjusted volume of interest
